# Ebola Virus RNA Stability in Human Blood and Urine in West Africa’s Environmental Conditions 

**DOI:** 10.3201/eid2202.151395

**Published:** 2016-02

**Authors:** Frédéric Janvier, Deborah Delaune, Thomas Poyot, Eric Valade, Audrey Mérens, Pierre E. Rollin, Vincent Foissaud

**Affiliations:** Hôpital d’Instruction des Armées Sainte Anne, Toulon, France (F. Janvier);; Centre de Traitement des Soignants, Conakry, Guinea (F. Janvier, D. Delaune, V. Foissaud);; Hôpital d’Instruction des Armées Bégin, Saint Mandé, France (D. Delaune, A. Mérens);; Institut de Recherche Biomédical des Armées, Brétigny-sur-Orge, France (T. Poyot, E. Valade);; Centers for Disease Control and Prevention, Atlanta, Georgia, USA (P.E. Rollin);; Hôpital d’Instruction des Armées Percy, Clamart, France (V. Foissaud)

**Keywords:** Ebola virus, RNA stability, human samples, rRT-PCR, viruses, West Africa, blood, urine, Ebola

## Abstract

We evaluated RNA stability of Ebola virus in EDTA blood and urine samples collected from infected patients and stored in West Africa’s environmental conditions. In blood, RNA was stable for at least 18 days when initial cycle threshold values were <30, but in urine, RNA degradation occurred more quickly.

Real-time reverse transcription PCR (rRT-PCR) has become the standard diagnostic tool for patients infected by Ebola virus (EBOV) (*1**,**2*). Control of the current outbreak and proper management of patients cannot be achieved without laboratory testing. In the field in West Africa and notably in Guinea, most Ebola treatment units are located near (often in the same compound) a laboratory that can process collected samples within hours. However, most healthcare centers or dispensaries lack this diagnostic capability and cannot ensure rapid testing of samples from patients suspected of having Ebola. 

Few laboratory studies have investigated Ebola virus RNA stability over time in collected samples. The period of stability has been estimated to be 5–14 days for virus suspensions on solid surfaces kept in darkness or for virus-spiked human blood or naturally infected, nonhuman primate blood stored under simulated tropical conditions (3–5). A recently published study found that viral RNA was consistently detectable in blood of cynomolgus macaques until 3 weeks after euthanasia (*6*). According to an anecdotal report, Ebola virus was isolated in blood samples stored for a month at room temperature (P.E. Rollin, pers. comm.). Such reports of sample stability must be considered for organizing collection and movement of samples in the field. In addition, quantification (copies/mL) or cycle threshold (C_t_) determination of EBOV RNA for estimating viral load has been shown to be a major prognosis marker in affected patients (*1**,**7**,**8*). C_t_ value, which can be used to perform RNA semiquantification, is not an absolute quantification, which necessitates use of a calibration curve. Moreover, C_t _value does not reflect viral viability but only presence of RNA in samples. By using C_t_ determination, we attempted to assess the stability of EBOV RNA in EDTA plasma (widely used in the field) and in urine from infected patients in the environmental conditions (i.e., air temperature and humidity) of West Africa. 

## The Study

Our study was conducted in the Laboratory of the Centre de Traitement des Soignants in Conakry, Guinea, during the Ebola outbreak in Guinea during January–March 2015. Establishing a Biosafety Level 4 (BSL-4) laboratory in Guinea was not possible. To enable biologists to work safely, a BSL-3 laboratory with a class-3 biologic-safety cabinet and single-use personal protective equipment were used (*9*).

We measured EBOV RNA stability in blood and urine samples from 7 case-patients with laboratory-confirmed Ebola. Twelve blood samples from the 7 case-patients were obtained by venipuncture by using Vacutainer tubes containing EDTA (BD Vacutainer; Becton Dickinson, Franklin Lakes, NJ, USA). Thirteen urine samples from the 7 case-patients were collected in individual sterile receptacles (BD Vacutainer Urine Collection Cup; Becton Dickinson).

Samples were immediately transferred to the laboratory. Upon arrival, samples were processed by centrifugation (at 4,000 rpm for 15 min) and stored at room temperature (22°C–29°C; 50%–80% humidity) for 18–30 days in a BSL-3 laboratory. At regular intervals (i.e., generally every other day), viral RNA was extracted from 100 μL of undiluted initial plasma or urine (QIAmp Viral RNA Mini Kit; QIAGEN, Valencia, CA, USA). To manage the limited volume of initial sample, our protocol used 100 μL of sample, less than manufacturer’s recommendations of 140 μL. We therefore modified the volume of lysis buffer (Buffer AVL; QIAGEN) to 400 μL to maintain the manufacturer’s recommended ratio of 1:4. We performed 40 cycles of rRT-PCR tests for detecting EBOV by using a commercially available kit (RealStar Filovirus Screen RT-PCR Kit 1.0; Altona Diagnostic GmbHh, Hamburg, Germany) (*10*). Results were expressed as C_t_ values, which are inversely proportional to the quantity of viral RNA in samples.

For each sample, extraction control and amplification of the positive control gave expected values; no inhibition of amplification occurred. At admission, all 7 case-patients had blood C_t _values <30.0 (mean 21.0, 95% CI 19.5–22.5). For the 12 blood samples studied (Figure, panel A), C_t_ values ranged from 18.3 to 35.0. When the initial C_t_ value was <30.0, C_t_ values were stable for up to 18 days after collection (day 0 mean 20.95, 95% CI 19.1–22.7; day 18 mean 21.9, 95% CI 19.9–23.8). For 2 samples with initial C_t_ values of 24.0 and 25.0, RNA was undetectable by days 28 and 22, respectively. For 3 samples collected from convalescent patients with C_t_ values ranging from 30.0 to 32.0, RNA was detected until day 4. For samples with C_t_ >32.0, RNA was detected on the initial test only.

**Figure:**
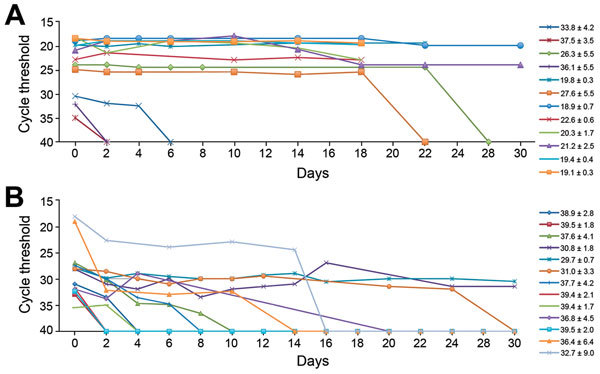
Stability of Ebola virus RNA in A) EDTA plasma and B) urine samples from patients in Guinea, as measured by real-time reverse transcription PCR. EDTA plasma and urine were processed immediately after receipt at a laboratory and left at room temperature (22°C–29°C; 50%–80% relative humidity) for various periods before the PCR analysis. Average cycle threshold values ± SD for individual samples are shown.

In the 13 urine samples (Figure, panel B), initial C_t_ values had a range of 18.2–35.5 (mean 28.6, 95% CI 25.7–31.5). RNA in urine was undetectable by days 10 and 14 for initial C_t_ counts of 19.0 and 18.0, respectively. For equivalent C_t_ values for blood, RNA was detectable for at least 18 days.

For urine samples with initial C_t _values >30.0, rRT-PCR results were negative within 2–4 days. However, urine results were inconsistent; 3 urine samples from the same patient had initial C_t_ values of 27.0–28.0 and were detectable for 24–30 days. 

These results from human samples taken during the Ebola outbreak in Guinea are similar to reported data for postmortem samples from cynomolgus macaques; for those samples, RNA was detectable for a few weeks after death (*6*). Our results indicate that EBOV RNA is stable in EDTA plasma samples collected and tested in the environmental conditions of West Africa. In the early phase of Ebola disease, blood sampling is probably more sensitive and reliable than oral swabbing and should be used whenever possible (*11**,**12*). Plasma samples can be tested for up to 18 days after collection, even if stored at ambient temperature, and positive results can still be reported. However, negative results may be false negative (in case of samples with low viral load) and should be reported as invalid; retesting should be performed on a new blood sample. 

Urine does not seem useful for initial diagnosis of Ebola disease. Even if collection is easy, detectable viral RNA appears in the urine of infected patients at a later stage of the disease than in blood, and C_t_ values are higher (*13**,**14*). The presence of protease, RNase, or bacteria and the absence of proteins that stabilize the virus and RNA in urine may explain the rapid degradation of viral RNA and thus the limited usefulness of this type of biologic sample.

This study is subject to limitations. First, our analysis was performed with samples that were centrifuged (i.e., plasma), according to manufacturer’s instructions and our laboratory protocol. No analysis was performed with noncentrifuged blood. For samples collected in facilities with no laboratory nearby, centrifugation before transport would not be possible and stability of viral RNA in these samples could have slightly different results than those found in this study. In such scenarios, for accurate comparisons of the stability of viral RNA over time, all tests would need to be performed on noncentrifuged blood, not on plasma. Second, the limited volume of collected samples did not enable us to perform tests in duplicate or at a frequency of every other day for 30 days for every sample.

## Conclusions

Our study was performed in real conditions (i.e., with samples from infected patients during the Ebola outbreak in Guinea in an Ebola treatment center laboratory there) and shows that EBOV RNA is stable in EDTA plasma. The development of practices for safe collection in the field and transport within a few hours or days to a local or national reference laboratory equipped with rRT-PCR capabilities seems feasible and will offer a more sustainable strategy for laboratory diagnosis and surveillance during and after the current outbreak.
